# Supplementary material for: Delivery of CRISPR/Cas9 Plasmid DNA by Hyperbranched Polymeric Nanoparticles Enables Efficient Gene Editing
Source: Cells. 2022 Dec 30;12(1):156. doi: 10.3390/cells12010156 (PMC9818138; doi:10.3390/cells12010156)

Supplementary Figure S1. TEM image of double-shell assembly of HP-25k polyplex upon addition of plasmid DNA (black).

HP-25K + pX458 (9.3 kb)

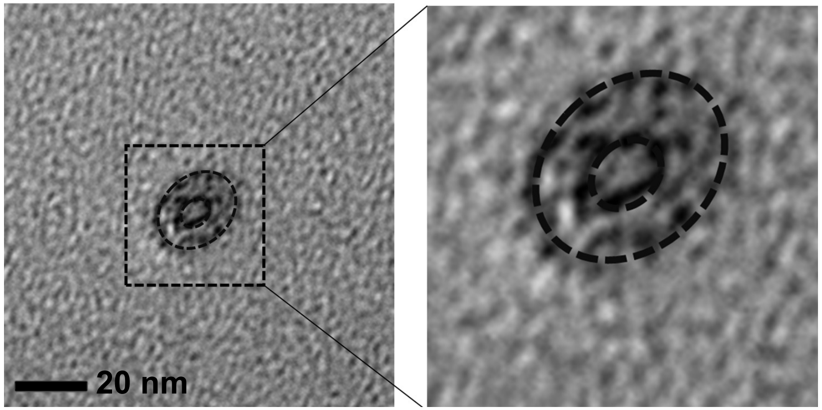

Supplementary Figure S2. Gel retardation assay of HP-800 packaged pmCherry-N1 pDNAs at different N/P ratios.

HP-800 + pmCherry-N1 (4.7 kb)

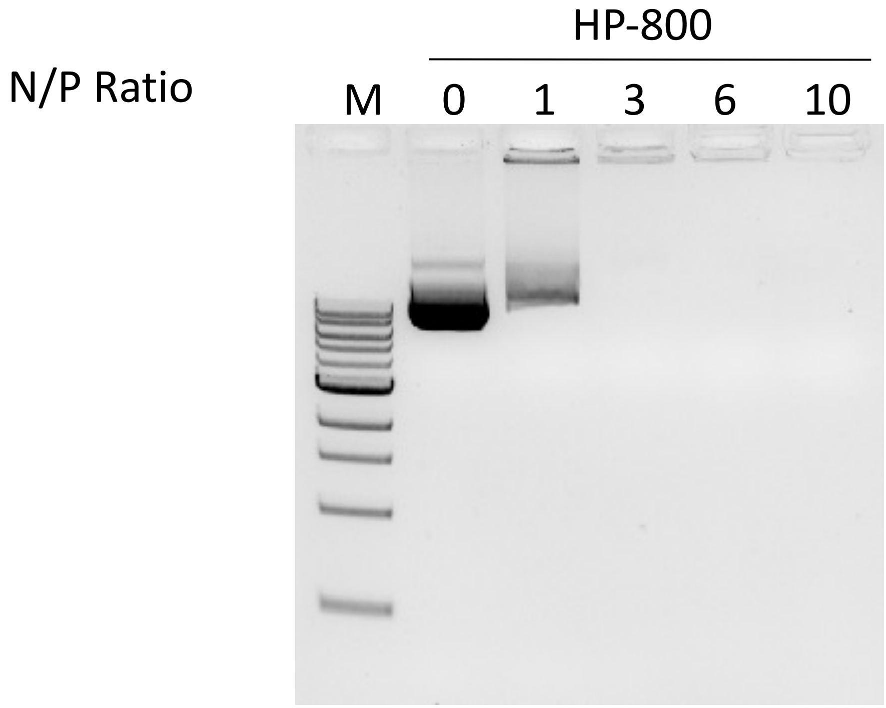

Supplementary Figure S3. FITC labeled HP-25K are efficiently taken up by AML12 (A) and HepG2 cells (B). Left, representative flow cytometry results. Right, summary of flow cytometry results.

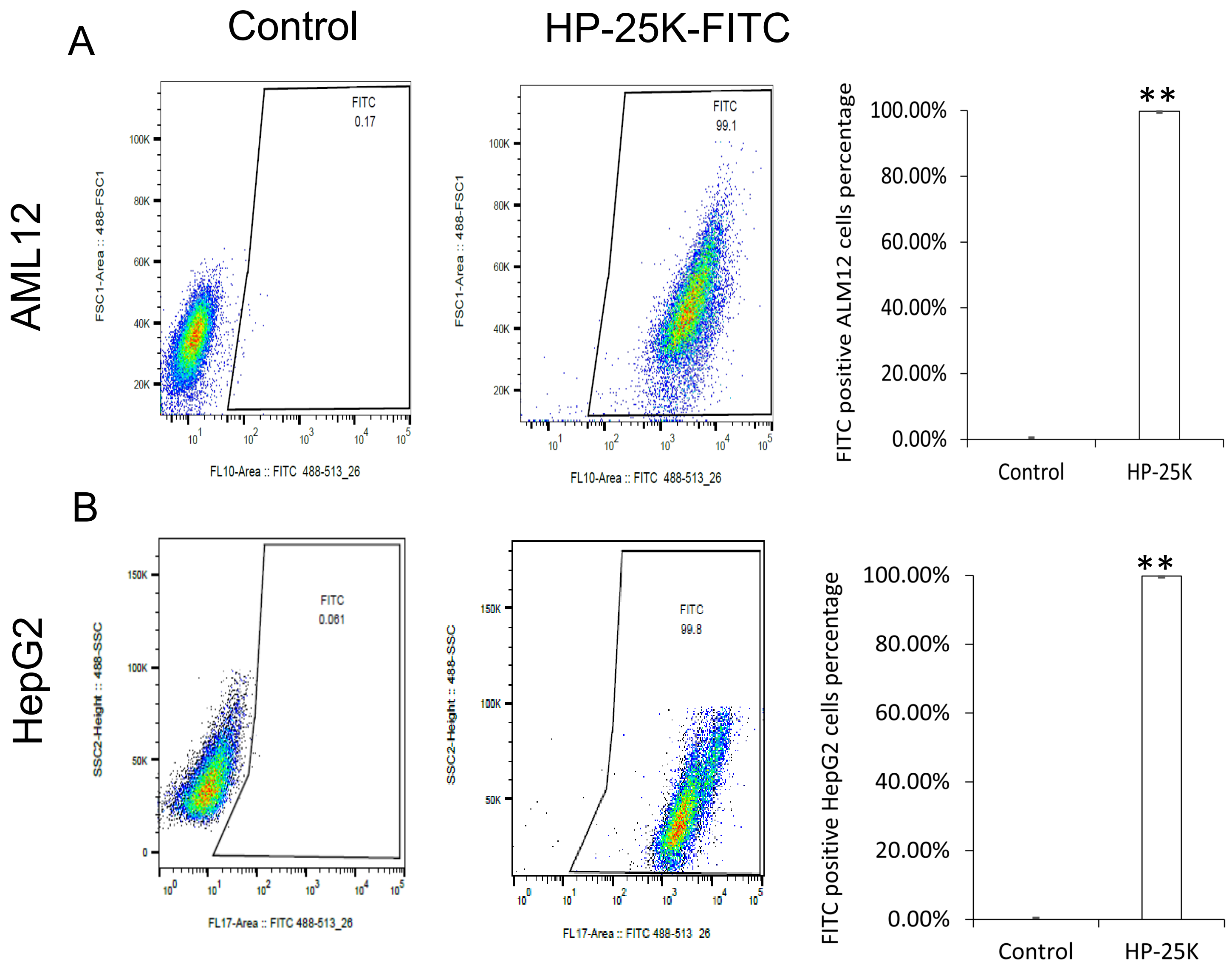

Supplementary Figure S4. HP-25K mediated delivery of pcDNA3-eGFP-LIC to Ad293 (A), Hela cells (B) or pmCherry-N1 to AML12 cells (C) lead to robust reporter gene expression.

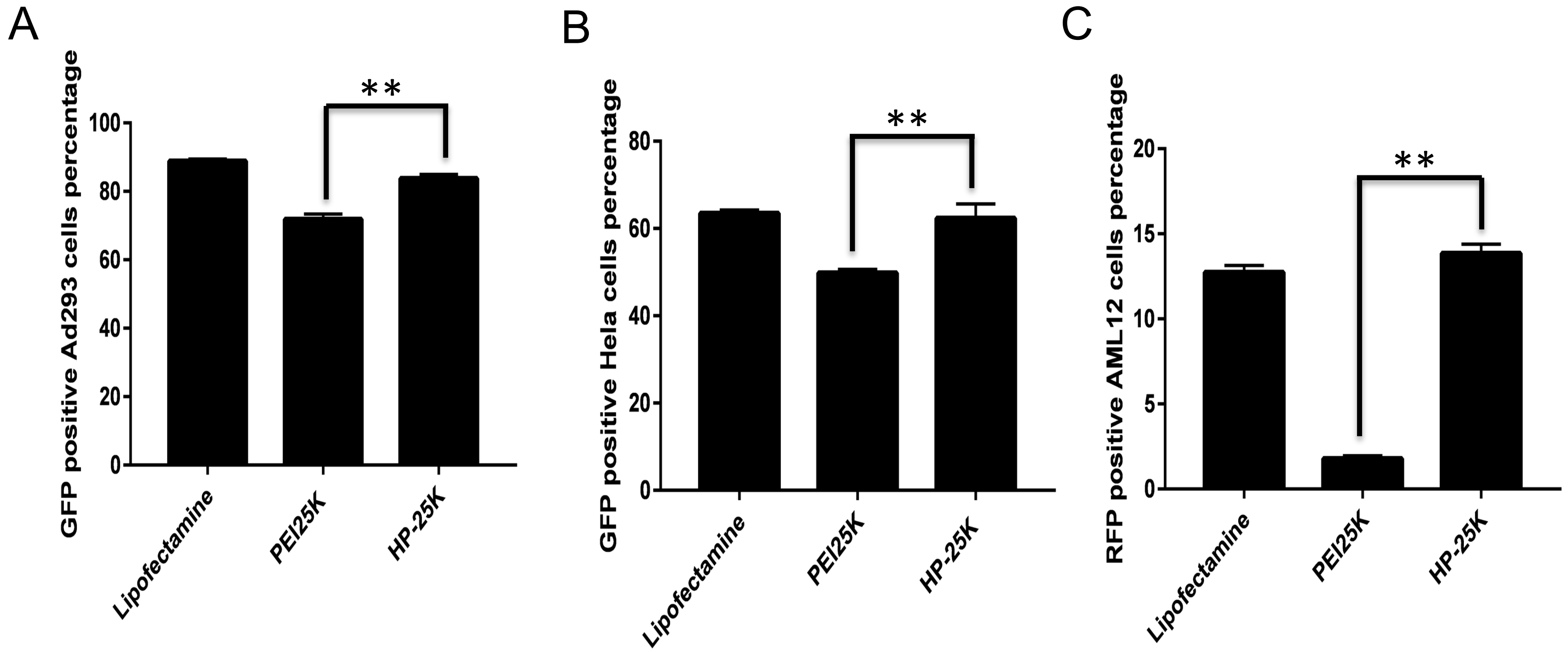

Supplement: Supplementary file 1 [file cells-12-00156-s001.zip › cells-2101787-supplementary.pdf]
